# Cardiac CT in Infective Aortic Prosthetic Valve Endocarditis

**DOI:** 10.5334/jbsr.3991

**Published:** 2025-07-03

**Authors:** Marwan Nadiri, Ana Falticeanu, Olivier Lebecque

**Affiliations:** 1 Université catholique de Louvain, CHU UCL Namur, Department of Radiology, 1 Avenue Dr G Thérasse, 5530 Yvoir, Belgium

**Keywords:** Endocarditis, prosthetic valve, cardiac CT

## Abstract

*Teaching point:* Cardiac computed tomography angiography is particularly valuable in patients with prosthetic valve infective endocarditis who have contraindications to transesophageal echocardiography (TOE), or when TOE is limited by heavy calcifications or the prosthetic valve, offering at least equivalent sensitivity for detecting abscesses and pseudoaneurysms.

## Case History

A 71‑year‑old male with a bioprosthetic aortic valve replacement, since 2017, developed persistent fever and was diagnosed with *Enterococcus faecalis* bacteremia following a recent urinary procedure. Transesophageal echocardiography (TOE) demonstrated a large sessile vegetation (2.6 × 1.5 cm) on the non‑coronary cusp of the bioprosthetic aortic valve – findings consistent with prosthetic valve infective endocarditis (IE), with no evidence of paravalvular abscess. Abdominal CT showed a small renal infarction, suggesting septic embolization.

**Figure 1:**
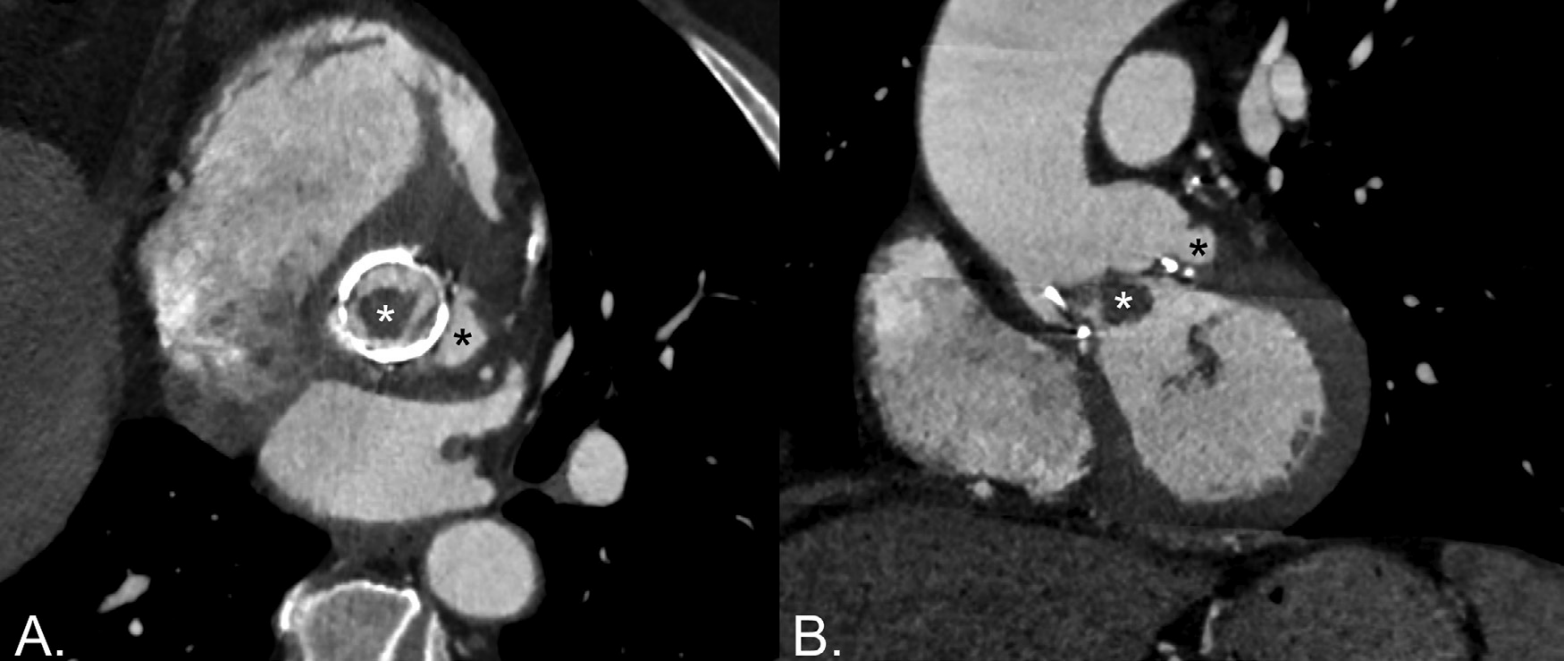
Multiplanar reconstructions showing a large vegetation (white asterisk) and a perivalvular pseudoaneurysm (black asterisk).

Cardiac computed tomography angiography (CCTA) demonstrated a nodular low‑attenuation lesion along the bioprosthetic leaflets, consistent with the large vegetation seen on TOE, and a left perivalvular contrast‑filled saccular outpouching, consistent with a pseudoaneurysm (Figure 1). ^18^F‑FDG PET/CT (PET) was considered, but the patient ultimately underwent urgent valve replacement. Intraoperative findings correlated with imaging results, revealing a friable mass attached to the prosthetic valve—corresponding to the vegetation identified on imaging—and a left subvalvular cavity consistent with the pseudoaneurysm seen on CCTA. No abscess or dehiscence was observed. The infected valve was replaced with a new bioprosthesis, and cultures of the excised valve yielded *E. faecalis*. The postoperative course and clinical follow‑up were unremarkable.

## Comments

The diagnosis of IE relies on a combination of clinical, microbiological, and imaging findings. Imaging has a key role in the modified Duke criteria, where it is considered a major criterion – particularly for diagnosing prosthetic valve IE – on par with microbiological evidence. Similarly, the 2023 recommendations from the European Society of Cardiology emphasize the importance of imaging, including not only TOE but also the CCTA and PET for suspected prosthetic valve IE [1].

ECG‑synchronized cardiac CT examination with thin‑section reconstruction does not replace echocardi‑ography, but it is at least equivalent to TOE in detecting abscesses and pseudoaneurysms. An abscess in IE is a perivalvular cavity with necrosis and purulent material. At CCTA, it is characterized by a low‑attenuation central necrotic component with a peripheral contrast‑enhancing rim. Phlegmon or early abscess formation can appear as soft tissue thickening. A pseudoaneurysm appears as a perivalvular contrast‑filled cavity, usually with a visible direct connection with the aortic root or cardiac chambers. Combining CCTA and TOE further enhances the diagnostic sensitivity. CCTA is particularly valuable in patients with contraindications to TOE or when TOE is suboptimal (i.e. due to heavy calcifications or the presence of prosthetic valves). TOE remains superior for identifying small vegetations (<10 mm), valvular leaflet perforations, and perivalvular leaks, but CCTA may still be useful in demonstrating these findings when TOE is either inconclusive or contraindicated [2].

PET has recently emerged as both an alternative and a complementary imaging modality in the diagnosis of IE. It is recommended in cases of possible prosthetic valve IE to help detect valvular lesions and support the diagnosis. PET demonstrates higher sensitivity for prosthetic valve IE and cardiac implantable electronic device infections compared to native valve IE. Its whole‑body imaging capability allows for the identification of additional infectious sites, such as the primary source of infection or septic emboli. PET can also reveal increased splenic and bone marrow activity that may represent indirect signs of IE [1].

TOE remains the first‑line imaging modality when IE is suspected, but CCTA and PET are increasingly used in the diagnosis and management of patients with IE. Recognizing typical imaging findings enhances the role of these advanced imaging tests in clinical practice when the diagnosis of IE is complicated or uncertain.
